# Psychological determinants of soil conservation behavior among farmers: conceptual drawbacks and alternative research approaches

**DOI:** 10.3389/fpsyg.2025.1489955

**Published:** 2025-05-12

**Authors:** Alexander Neaman

**Affiliations:** Facultad de Ciencias Agronómicas, Universidad de Tarapacá, Arica, Chile

**Keywords:** land degradation, attitude, action, emotion, protection, soil, engagement, behavior

## Introduction

Environmental degradation, deeply intertwined with human activities (Schultz, 2011), requires multifaceted solutions. While technological and legislative measures are crucial, exploring psychological factors is equally important. In the realm of soil conservation, a significant knowledge gap persists regarding the psychological motivations that influence farmers' adoption of sustainable practices.

Soil management is a crucial component of the life support system for human civilization (Brevik et al., 2018). The collapse of several ancient civilizations due to inadequate soil management (Montgomery, 2012) underscores the necessity of investigating the psychological factors affecting farmers' adoption of soil conservation practices to inform evidence-based strategies for promoting sustainable soil management.

Environmental psychology, which examines pro-environmental behaviors in general (Gatersleben, 2019), provides a valuable framework for understanding the complex interactions between humans and the environment. Soil conservation behavior research is a more specialized area of study that specifically investigates the actions farmers take to minimize soil degradation and adopt environmentally sustainable farming practices (Bijani et al., 2019).

In the author's opinion, current research on soil conservation behavior is characterized by several conceptual limitations:

1) A significant proportion of studies on soil conservation behavior focus on farmers' intentions rather than behaviors, neglecting the potential discrepancy between stated intentions and actual conservation practices.
2) Current approaches to understanding behavior change often prioritize cognitive variables (e.g., knowledge), but neglect the influence of emotional and affective factors, which also play a crucial role.
3) The effectiveness of different educational approaches in promoting soil conservation is rarely evaluated.
4) The availability of scales to measure soil conservation behaviors is limited, and existing scales often focus on specific behaviors rather than broader representation of a farmer's predisposition to preserve soil resources.
5) Mediation models are frequently based on cross-sectional data, which can be problematic when the directionality of the relationships is unclear.
6) Longitudinal studies are scarce in soil conservation behavior research, and those that do exist often focus on farmers' knowledge of soil science rather than actual behavior change.

The subsequent sections provide an in-depth examination of these conceptual limitations, accompanied by proposed alternative approaches aimed at addressing these gaps and improving the understanding of soil conservation behavior.

## Discussion

### The importance of focusing on actual behaviors

Behavioral intentions, or a person's readiness to perform a behavior, have been the focus of numerous studies on soil conservation (Lu et al., 2022; Rabinovich et al., 2022). These studies have examined factors such as willingness to support a policy, participate in a program, or engage in a conservation practice. However, it is well established that intentions do not always translate into actual behaviors, a phenomenon known as the “intention-behavior gap” (Fishbein and Ajzen, 2010).

Given this discrepancy, several scholars have emphasized the need to use behavioral willingness with caution (Floress et al., 2018). While behavioral intentions can be useful for program development, for instance, by focusing efforts on practices with positive willingness (Yeboah et al., 2015), the ultimate goal should be to shift respondents from intention to actual behavior change. In the opinion of the author, any such behavior change should be carefully quantified at the end of the program to assess its effectiveness.

### Leveraging emotions for behavior change

Behavior change is a complex, multi-faceted process that unfolds gradually (Prochaska and Velicer, 1997). The stage model of self-regulated behavioral change (SSBC) provides a valuable framework for understanding this process, outlining four distinct stages through which individuals progress as they modify their behaviors (Bamberg, 2013). Bamberg and Schulte (2019) emphasize tailoring interventions to the specific stage of change an individual has reached. In the early stages (pre-decision and pre-action) interventions should focus on raising awareness of the issue and fostering a sense of personal responsibility. Providing information about alternative behaviors and their associated benefits and drawbacks can be beneficial at this stage. In the later stages (action and post-action) individuals require support in developing detailed implementation plans to translate their intentions into action and maintain the changed behavior (Gollwitzer, 1999).

While the SSBC model by Bamberg (2013) offers a cognitive perspective on behavior change, it is essential to acknowledge the significant role of emotions (Taufik and Venhoeven, 2019). As Williamson and Thulin (2022) point out, leveraging human emotions for behavior change remains an underutilized approach compared to other strategies, such as those focused on social norms.

Emotions play a pivotal role in shaping individuals' relationships with the natural world, including soils. Research has identified various emotions associated with nature and soils, such as fascination (Lumber et al., 2017), respect (Eisenberg, 2013; Quintriqueo et al., 2014), and interest (Kals et al., 1999).

Scholars have proposed the concept of “connection to soil,” which refers to the emotional bond that farmers develop with the land they cultivate (Charzynski et al., 2022). While this concept may appear similar to the more general “connection to nature” (Mayer and Frantz, 2004), the author argues that it represents a distinct and more targeted construct. Similar to the distinction between general and specific attitudes in social psychology (Bamberg, 2003; Kroesen and Chorus, 2018), a general “connection to nature” may not directly translate into specific soil conservation behaviors.

By focusing on the specific emotional bond with soil, research can gain a more nuanced understanding of farmer behavior. Recent research has demonstrated that connection to soil is a correlate of soil conservation behavior among farmers (Burnham et al., 2023). This suggests that fostering farmers' emotional attachment to the land they manage may be a promising avenue for promoting the adoption of soil conservation practices.

### Evaluating educational approaches for promoting soil stewardship

At the university level, students enrolled in agricultural classes typically receive instruction during the semester, yet the impact of this teaching on their sense of soil care remains largely unquantified. Furthermore, there is uncertainty regarding the most effective educational approaches to foster desired shifts in the students' soil care attitudes and behaviors (Neaman et al., 2021).

As Field et al. (2017) point out, soil science teaching often prioritizes knowledge necessary to meet the demands of the agricultural industry. Despite students being the future stewards of the soil, our understanding of how to effectively promote soil care in them is surprisingly limited. This issue is exacerbated by the increasing urbanization of the world, which often leads to many agriculture students lacking a strong connection to the soil (Hartemink et al., 2014).

One promising approach to stimulate changes conducive to soil care is through pedagogical methods designed to instill a sense of wonder and fascination for the subject matter (Otto et al., 2020), in this case, the soil itself. Recognizing the importance of emotional engagement in learning (Pooley and O'connor, 2000), various methods to foster a deeper connection to soil can be employed. Inspired by Hartemink et al. (2014), classes can include activities using natural earth materials, like creating soil paintings. Instructors can also integrate emotionally charged narratives and storytelling techniques (Brevik et al., 2022a,b) to cultivate a sense of wonder and appreciation for soil.

By combining this diverse, emotionally engaging approach with core soil science knowledge, the aim is to create a relatable and engaging learning experience that fosters emotional connections and a deeper understanding of soil conservation practices. However, the precise effect of different educational approaches on students' sense of soil care needs to be carefully evaluated (Neaman et al., 2021). Consequently, further research in this area is imperative to inform the development of effective soil science education curricula.

### Expanding the measurement of soil conservation behaviors

Reliable and comprehensive measurement tools are essential for establishing theoretical foundations and testing research hypotheses in the field of soil conservation behavior. However, this area of research has been hampered by a lack of well-developed scales for collecting empirical data on farmers' soil conservation behaviors.

The existing scales for assessing farmers' soil conservation behavior are often designed to measure the perspectives of particular types of farmers, such as those cultivating rice (Bijani et al., 2017). This limited scope raises concerns about the generalizability of the findings.

Another crucial issue is the restricted focus on specific behaviors employed to gauge farmers' engagement, as exemplified by the soil conservation behavior scale introduced by Borkhani et al. (2023). This narrow focus overlooks the substantial variation in individual living situations, which may offer unique behavioral opportunities that differ from farmer to farmer and from circumstance to circumstance. Farmers possess a varied range of behavioral options that they can choose to apply, such as adopting organic agriculture (Bouttes et al., 2019) instead of exclusively focusing on minimum tillage (Dedecker et al., 2022).

To address this limitation, future studies should consider a multi-domain approach to capturing farmers' soil conservation behaviors, as demonstrated by the scale developed by Burnham et al. (2023). This broader representation of a farmer's predisposition to preserve soil resources, regardless of the specifics of each practice, shifts the emphasis from individual behaviors to the farmer's overall view on agriculture and the extent to which they adopt soil conservation practices.

By employing a multi-domain approach, researchers can better understand the diverse motivations and decision-making processes that underlie farmers' soil conservation behaviors. This, in turn, can inform the development of more effective interventions and policies to promote sustainable soil management practices.

### Limitations of mediation models based on cross-sectional data

Mediation models are commonly used in environmental psychology to examine the relationships between different constructs (Otto et al., 2021). However, this approach relies on the assumption of known causal pathways, which can be problematic when the directionality of the relationships is unclear.

For instance, regarding the correlation between an individual's connection to nature and their ecological behavior, many researchers have supported the idea that the causal pathway is from “connection to nature” to “ecological behavior” rather than the other way around (Steg and Vlek, 2009; Whitburn et al., 2020). However, a study by Kaiser et al. (2014) using longitudinal survey data in a cross-lagged structural equation model found no evidence of causation between these two variables. Similarly, the “knowledge-deficit theory” proposed by Schultz (2002) suggests that a lack of environmental knowledge can hinder environmental action. Yet, recent studies have suggested that environmental knowledge may be a consequence, rather than a cause, of ecological behavior (Taube et al., 2021; Baierl et al., 2022).

Despite the absence of definitive evidence for the causal relationships between different constructs used in environmental psychology, mediation models based on cross-sectional data are still commonly employed in this field. This approach has been criticized (e.g., Maxwell et al., 2011) for its limitations in establishing causality.

To address this issue, longitudinal mediation analysis using a random intercept cross-lagged panel model (Lilly et al., 2023) might be a better approach for environmental psychology research, including studies on soil conservation behavior. This method allows for the examination of reciprocal relationships and the identification of causal pathways, providing more robust insights into the underlying mechanisms driving environmental behaviors.

### Toward a comprehensive approach to soil conservation interventions

Existing outreach programs in soil conservation often lack robust evaluation methods. Many studies focus solely on participation metrics, such as attendance (Lobry De Bruyn et al., 2017; Floress et al., 2018), while others primarily evaluate soil science knowledge gains, overlooking potential changes in attitudes and behaviors (Rejesus et al., 2012; Pan and Zhang, 2018). This limited scope undermines our understanding of the true impact of these interventions.

However, research from environmental psychology offers promising frameworks for developing more effective soil conservation interventions. Studies have demonstrated the success of interventions in fostering a stronger connection to nature among adults (Coughlan et al., 2022), which in turn has been linked to increased pro-environmental behaviors (Deville et al., 2021). In addition, longitudinal research shows that interventions can effectively enhance pro-environmental behavior (Otto and Pensini, 2017).

These findings highlight the need for a comprehensive, longitudinal approach to investigating the effectiveness of soil conservation interventions. Such studies should examine whether interventions can promote soil conservation by simultaneously strengthening farmers' connection to soil and improving their soil science knowledge. Recent pilot studies (Burnham et al., 2023; Neaman et al., 2024) have established robust measurement tools that provide a solid foundation for conducting this type of research across diverse farming populations.

By adopting a comprehensive, longitudinal research design, scholars can assess the intervention's effectiveness in promoting not only knowledge acquisition but also positive attitude shifts and, ultimately, behavioral changes toward sustainable soil conservation practices.

## References

[B1] Baierl T. M. Kaiser F. G. Bogner F. X. (2022). The supportive role of environmental attitude for learning about environmental issues. J. Environ. Psychol. 81:101799. 10.1016/j.jenvp.2022.101799

[B2] Bamberg S. (2003). How does environmental concern influence specific environmentally related behaviors? A new answer to an old question. J. Environ. Psychol. 23, 21–32. 10.1016/S0272-4944(02)00078-6

[B3] Bamberg S. (2013). Changing environmentally harmful behaviors: a stage model of self-regulated behavioral change. J. Environ. Psychol. 34, 151–159. 10.1016/j.jenvp.2013.01.002

[B4] Bamberg S. Schulte M. (2019). “Processes of Change,” in Environmental Psychology: An Introduction, eds. L. Steg and J.I.M. De Groot (Toronto, Canada: John Wiley & Sons), 307–318.

[B5] Bijani M. Ghazani E. Valizadeh N. Haghighi N. F. (2017). Pro-environmental analysis of farmers' concerns and behaviors towards soil conservation in central district of Sari County, Iran. Int. Soil Water Conserv. Res. 5, 43–49. 10.1016/j.iswcr.2017.03.001

[B6] Bijani M. Ghazani E. Valizadeh N. Haghighi N. F. (2019). Predicting and understanding farmers' soil conservation behavior in Mazandaran Province, Iran. J. Agric. Sci. Technol. 21, 1705–1719.

[B7] Borkhani F. R. Khaleghi B. Mirtorabi M. S. Mohammadi Y. (2023). Explaining farmers' pro-environmental behaviors toward plant, soil and water conservation in Iran: an application of value-belief-norm theory. Int. J. Environ. Sci. Technol. 20, 2539–2550. 10.1007/s13762-022-04568-z

[B8] Bouttes M. Darnhofer I. Martin G. (2019). Converting to organic farming as a way to enhance adaptive capacity. Org. Agric. 9, 235–247. 10.1007/s13165-018-0225-y

[B9] Brevik E. C. Hannam J. Krzic M. Muggler C. Uchida Y. (2022a). The importance of soil education to connectivity as a dimension of soil security. Soil Sec. 7:100066. 10.1016/j.soisec.2022.100066

[B10] Brevik E. C. Homburg J. A. Sandor J. A. (2018). “Soils, climate, and ancient civilizations,” in Climate Change Impacts on Soil Processes and Ecosystem Properties, eds. *W*. Horwath and Y. Kuzyakov. (Oxford, UK: Elsevier), 1–28. 10.1016/B978-0-444-63865-6.00001-6

[B11] Brevik E. C. Krzic M. Muggler C. Field D. Hannam J. Uchida Y. (2022b). Soil science education: a multinational look at current perspectives. Nat. Sci. Educ. 51:e20077. 10.1002/nse2.20077

[B12] Burnham E. Zabel S. Navarro-Villarroel C. Ermakov D. S. Castro M. Neaman A.. (2023). Enhancing farmers' soil conservation behavior: beyond soil science knowledge. Geoderma 437:116583. 10.1016/j.geoderma.2023.116583

[B13] Charzynski P. Urbanska M. Capra G. F. Ganga A. Holmes P. Szulczewski M.. (2022). A global perspective on soil science education at third educational level; knowledge, practice, skills and challenges. Geoderma 425:116053. 10.1016/j.geoderma.2022.116053

[B14] Coughlan A. Ross E. Nikles D. De Cesare E. Tran C. Pensini P. (2022). Nature guided imagery: An intervention to increase connectedness to nature. J. Environ. Psychol. 80, 1–8. 10.1016/j.jenvp.2022.101759

[B15] Dedecker J. Malone T. Snapp S. Thelen M. Anderson E. Tollini C.. (2022). The relationship between farmer demographics, social identity and tillage behavior: evidence from Michigan soybean producers. J. Rural Stud. 89, 378–386. 10.1016/j.jrurstud.2022.01.001

[B16] Deville N. V. Tomasso L. P. Stoddard O. P. Wilt G. E. Horton T. H. Wolf K. L.. (2021). Time spent in nature is associated with increased pro-environmental attitudes and behaviors. Int. J. Environ. Res. Public Health 18:7498. 10.3390/ijerph18147498 34299948 PMC8305895

[B17] Eisenberg A. (2013). Aymara Indian Perspectives on Development in the Andes. Tuscaloosa, AL: University of Alabama Press.

[B18] Field D. J. Yates D. Koppi A. J. Mcbratney A. B. Jarrett L. (2017). Framing a modern context of soil science learning and teaching. Geoderma 289, 117–123. 10.1016/j.geoderma.2016.11.034

[B19] Fishbein M. Ajzen I. (2010). Predicting and Changing Behavior: The Reasoned Action Approach. New York: Psychology Press. 10.4324/9780203838020

[B20] Floress K. Reimer A. Thompson A. Burbach M. Knutson C. Prokopy L.. (2018). Measuring farmer conservation behaviors: challenges and best practices. Land Use Policy 70, 414–418. 10.1016/j.landusepol.2017.11.030

[B21] Gatersleben B. (2019). “Measuring Environmental Behaviour,” in Environmental Psychology: An Introduction, eds. L. Steg and J. I. M. De Groot (Toronto, Canada: John Wiley & Sons), 157–166.

[B22] Gollwitzer P. M. (1999). Implementation intentions: strong effects of simple plans. Am. Psychol. 54, 493–503. 10.1037/0003-066X.54.7.493

[B23] Hartemink A. E. Balks M. R. Chen Z. S. Drohan P. Field D. J. Krasilnikov P.. (2014). The joy of teaching soil science. Geoderma 217, 1–9. 10.1016/j.geoderma.2013.10.016

[B24] Kaiser F. G. Brügger A. Hartig T. Bogner F. X. Gutscher H. (2014). Appreciation of nature and appreciation of environmental protection: how stable are these attitudes and which comes first? Eur. Rev. Appl. Psychol. 64, 269–277. 10.1016/j.erap.2014.09.001

[B25] Kals E. Schumacher D. Montada L. (1999). Emotional affinity toward nature as a motivational basis to protect nature. Environ. Behav. 31, 178–202. 10.1177/00139169921972056

[B26] Kroesen M. Chorus C. (2018). The role of general and specific attitudes in predicting travel behavior—a fatal dilemma? Travel Behav. Soc. 10, 33–41. 10.1016/j.tbs.2017.09.004

[B27] Lilly K. J. Sibley C. G. Osborne D. (2023). Examining the indirect effect of income on well-being via individual-based relative deprivation: longitudinal mediation with a random intercept cross-lagged panel model. Int. J. Psychol. 59, 368–377. 10.1002/ijop.13097 38018263

[B28] Lobry De Bruyn L. L. Jenkins A. Samson-Liebig S. (2017). Lessons learnt: sharing soil knowledge to improve land management and sustainable soil use. Soil Sci. Soc. Am. J. 81, 427–438. 10.2136/sssaj2016.12.0403

[B29] Lu J. Ranjan P. Floress K. Arbuckle J. G. Church S. P. Eanes F. R.. (2022). A meta-analysis of agricultural conservation intentions, behaviors, and practices: Insights from 35 years of quantitative literature in the United States. J. Environ. Manag. 323:116240. 10.1016/j.jenvman.2022.116240 36261983

[B30] Lumber R. Richardson M. Sheffield D. (2017). Beyond knowing nature: contact, emotion, compassion, meaning, and beauty are pathways to nature connection. PLoS ONE 12:e0177186. 10.1371/journal.pone.0177186 28486515 PMC5423657

[B31] Maxwell S. E. Cole D. A. Mitchell M. A. (2011). Bias in cross-sectional analyses of longitudinal mediation: partial and complete mediation under an autoregressive model. Multivariate Behav. Res. 46, 816–841. 10.1080/00273171.2011.606716 26736047

[B32] Mayer F. S. Frantz C. M. P. (2004). The connectedness to nature scale: a measure of individuals' feeling in community with nature. J. Environ. Psychol. 24, 503–515. 10.1016/j.jenvp.2004.10.001

[B33] Montgomery D. R. (2012). Dirt: The Erosion of Civilizations. Berkeley, CA: University of California Press. 10.1525/9780520952119

[B34] Neaman A. Navarro-Villarroel C. Poblete-Ramos F. Lizardi N. Burnham E. Huerta-Salinas O.. (2024). Reconciling the soil stewardship paradox: knowledge without care, care without knowledge. Geoderma Regional 37:e00794. 10.1016/j.geodrs.2024.e00794

[B35] Neaman A. Stange C. Zabel S. Minkina T. M. Yanez C. Burnham E.. (2021). Teaching soil science: the impact of laboratory and field components on the knowledge and attitude toward soil. Rev. Bras. Ciênc. Solo 45:e0210040. 10.36783/18069657rbcs20210040

[B36] Otto S. Körner F. Marschke B. A. Merten M. J. Brandt S. Sotiriou S.. (2020). Deeper learning as integrated knowledge and fascination for science. Int. J. Sci. Educ. 42, 807–834. 10.1080/09500693.2020.1730476

[B37] Otto S. Pensini P. (2017). Nature-based environmental education of children: environmental knowledge and connectedness to nature, together, are related to ecological behaviour. Glob. Environ. Change 47, 88–94. 10.1016/j.gloenvcha.2017.09.009

[B38] Otto S. Pensini P. Zabel S. Diaz-Siefer P. Burnham E. Navarro-Villarroel C.. (2021). The prosocial origin of sustainable behavior: a case study in the ecological domain. Glob. Environ. Change Hum. Policy Dimens. 69:102312. 10.1016/j.gloenvcha.2021.102312

[B39] Pan D. Zhang N. (2018). The role of agricultural training on fertilizer use knowledge: a randomized controlled experiment. Ecol. Econ. 148, 77–91. 10.1016/j.ecolecon.2018.02.004

[B40] Pooley J. A. O'connor M. (2000). Environmental education and attitudes—emotions and beliefs are what is needed. Environ. Behav. 32, 711–723. 10.1177/0013916500325007

[B41] Prochaska J. O. Velicer W. F. (1997). The transtheoretical model of health behavior change. Am. J. Health Promot. 12, 38–48. 10.4278/0890-1171-12.1.38 10170434

[B42] Quintriqueo S. Quilaqueo D. Torres H. (2014). Contribution for the teaching of natural sciences: mapuche and school knowledge. Educação e Pesquisa 40, 965–982. 10.1590/S1517-97022014005000009

[B43] Rabinovich A. Zhischenko V. Nasseri M. Heath S. C. Laizer A. Mkilema F.. (2022). Informing versus generating a discussion: comparing two approaches to encouraging mitigation of soil erosion among maasai pastoralists. J. Environ. Psychol. 84:101885. 10.1016/j.jenvp.2022.101885

[B44] Rejesus R. M. Mutuc M. E. M. Yasar M. Lapitan A. V. Palis F. G. Truong T. N. C. (2012). Sending vietnamese rice farmers back to school: further evidence on the impacts of farmer field schools. Can. J. Agric. Econ. 60, 407–426. 10.1111/j.1744-7976.2011.01242.x

[B45] Schultz P. W. (2002). “Knowledge, information, and household recycling: examining the knowledge-deficit model of behavior change,” in New Tools for Environmental Protection: Education, Information, and Voluntary Measures, eds. T. Dietz and P.C. Stern (Washington, DC: National Academy Press), 67–82.

[B46] Schultz P. W. (2011). Conservation means behavior. Conserv. Biol. 25, 1080–1083. 10.1111/j.1523-1739.2011.01766.x 22070255

[B47] Steg L. Vlek C. (2009). Encouraging pro-environmental behaviour: an integrative review and research agenda. J. Environ. Psychol. 29, 309–317. 10.1016/j.jenvp.2008.10.004

[B48] Taube O. Ranney M. A. Henn L. Kaiser F. G. (2021). Increasing people's acceptance of anthropogenic climate change with scientific facts: is mechanistic information more effective for environmentalists? J. Environ. Psychol. 73:101549. 10.1016/j.jenvp.2021.101549

[B49] Taufik D. Venhoeven L. (2019). “Emotions and pro-environmental behavior,” in Environmental Psychology: An Introduction, eds. L. Steg and J. I. M. De Groot (Toronto, Canada: John Wiley & Sons), 189–197. 10.1002/9781119241072.ch19

[B50] Whitburn J. Linklater W. Abrahamse W. (2020). Meta-analysis of human connection to nature and proenvironmental behavior. Conserv. Biol. 34, 180–193. 10.1111/cobi.13381 31251416 PMC7027494

[B51] Williamson K. A. Thulin E. (2022). Leveraging emotion-behavior pathways to support environmental behavior change. Ecol. Soc. 27:27. 10.5751/ES-13363-270327

[B52] Yeboah F. K. Lupi F. Kaplowitz M. D. (2015). Agricultural landowners' willingness to participate in a filter strip program for watershed protection. Land Use Policy 49, 75–85. 10.1016/j.landusepol.2015.07.016

